# *In Vitro* Antibacterial Spectrum of* Sodium Selenite* against Selected Human Pathogenic Bacterial Strains

**DOI:** 10.1155/2016/9176273

**Published:** 2016-03-15

**Authors:** Mohammad Firoz Alam, Mohammed M. Safhi, Sivakumar Sivagurunathan Moni, Aamena Jabeen

**Affiliations:** ^1^Department of Pharmacology & Toxicology, College of Pharmacy, Jazan University, P.O. Box 114, Jazan 45142, Saudi Arabia; ^2^Division of Pharmaceutical Biotechnology, Department of Pharmaceutics, College of Pharmacy, Jazan University, P.O. Box 114, Jazan 45142, Saudi Arabia

## Abstract

The objective of this investigation was to predict the antibacterial properties of* sodium selenite* against selected human pathogens. A group of six human bacterial pathogens including* Staphylococcus aureus*,* Streptococcus pyogenes*,* Bacillus subtilis*,* Escherichia coli*,* Pseudomonas aeruginosa*, and* Klebsiella planticola* were utilized for screening. The spectrum of activity was qualified based on zone of inhibition. Our study demonstrated that* sodium selenite* exhibits a strong spectrum of activity against* Bacillus subtilis*,* Staphylococcus aureus*,* Escherichia coli*, and* Klebsiella planticola.* The spectrum of activity was compared with standard ciprofloxacin disc (5 *μ*g/disc) and observed to have satisfactory effect.

## 1. Introduction

*Sodium selenite* is an element that is reported to have an antioxidant property as well as potential anticancer activity [1, 2].* Sodium selenite* is also known as disodium salt of selenious acid which is colorless and a water soluble solid inorganic compound mainly used in the manufacturing of colorless glass [3]. Selenium was first identified as an essential trace element in mammals in 1997 and was known to be required for variety of functional Se-dependent proteins (selenoproteins) in most living organisms [4–6]. Selenium acts as a supplement for the enzyme glutathione peroxidase [7, 8], which is involved in the normal permeability of cell membranes, by removing H_2_O_2_ and preventing the production of lipid peroxides [9]. Certain selenium compounds have a catalytic property by producing Reactive Oxygen Species (ROS) through interaction with thiols, such as reduced glutathione, forming the glutathione selenide anion, GSSe [10]. ROS, including superoxide radical, hydrogen peroxide, and hydroxide radical, causes cellular damage such as DNA oxidation, lipid peroxidation, and protein oxidation [11]. The paradox of selenium (Se) is that it is both essential and toxic to living organism. Many of the earlier reports showed that the mechanism of selenium compounds is still unclear. The essentiality and toxicity of selenium in vertebrates generate the remarkable scientific research interest in this element. In this research work we proposed a model to screen the antibacterial properties of* sodium selenite* as very limited works have been reported to find out the spectrum of activity. Therefore, we are reporting the antibacterial properties of* sodium selenite* against the selected Gram positive and Gram negative human pathogenic bacteria.

## 2. Materials and Method

### 2.1. Preparation of* Sodium Selenite* 1% w/v Stock Solution

*Sodium selenite* was obtained from Sigma Aldrich, India. It was white powder and highly soluble in water. Working stock solution of 1% w/v of* sodium selenite* was prepared by dissolving in double distilled sterilized water. The 1% w/v* solution of sodium selenite* was clear and transparent.

### 2.2. Strains Used

Six bacterial strains* Staphylococcus aureus*,* Streptococcus pyogenes*,* Bacillus subtilis*,* Escherichia coli*,* Pseudomonas aeruginosa*, and* Klebsiella planticola* were isolated from clinical samples obtained from Jazan Hospital, Jazan. The stock cultures were subcultured and the working culture was assessed as 10^−6^ CFU/mL. Specified quantity of Muller Hinton agar was prepared and plated in aseptic condition. The agar well diffusion technique was followed to perform the antibacterial susceptibility test of* sodium selenite* and agar disc diffusion method was followed for standard ciprofloxacin disc (5 *μ*g/disc). After 24 h of incubation at 37°C the zone of inhibition was measured and tabulated.

### 2.3. Statistical Analysis

All the experiments were performed six times (*n* = 6) and the data were subjected to one way analysis of variance (ANOVA), and the level of significance is *P* < 0.001 using Graphpad Instat software system, USA. The test values were compared with standard drug values by using Tukey Kramer test (*post hoc* analysis).

## 3. Results

In this study we screened the antibacterial properties of* sodium selenite* against selected human pathogenic bacteria. The results are summarized in Table 1 demonstrating that 1% w/v solution of* sodium selenite* showed predominant activity against* Bacillus subtilis*,* Staphylococcus aureus*,* Escherichia coli*, and* Klebsiella planticola*. It is noteworthy that in our study the spectrum of antibacterial activity of* sodium selenite* has been proved against selected Gram positive and Gram negative human pathogenic bacteria of clinical origin. The statistical studies were also performed to compare the efficacy of* sodium selenite* among Gram positive and Gram negative bacteria, which is presented in Tables 2 and 3.  Table 2 explains the efficacy of* sodium selenite* among Gram positive and Gram negative bacteria. In general, the results were very clear where* sodium selenite* exhibited a wider spectrum of activity. However, predominant activity was observed in* Klebsiella planticola*,* Bacillus subtilis*,* Staphylococcus aureus*, and* Escherichia coli *which is displayed in Table 3.

## 4. Discussion

**Figure 1:**
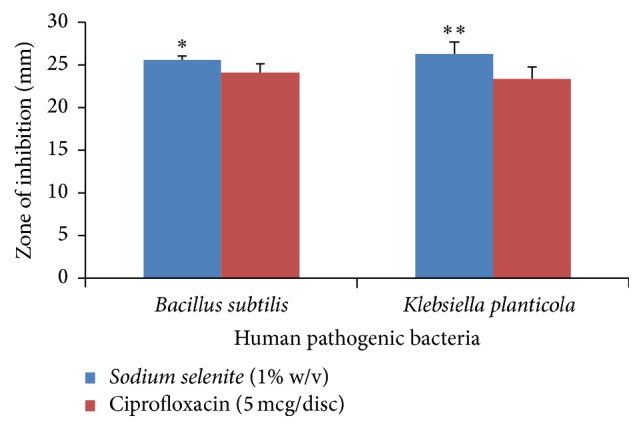
Significant spectrum of activity of* sodium selenite* when compared to standard ciprofloxacin, ^*∗*^ *P* < 0.05* sodium selenite* when compared to ciprofloxacin in* Bacillus subtilis* and ^*∗∗*^ *P* < 0.01* sodium selenite* when compared to ciprofloxacin in* Klebsiella planticola*.

*Sodium selenite* is used as a nutritional supplement in poultry feed to promote growth and prevent selenium deficiency diseases [12]. Studies have been reported that* sodium selenite* is having potential antineoplastic activity. However, exploitation of* sodium selenite* as a pharmaceutical agent is very limited. There are various studies which have been established to explore the anticancer property of* sodium selenite* due to its antioxidant properties. But very limited researchers have concentrated on antibacterial properties of* sodium selenite*. In our earlier study we have reported that 1% w/v solution of sodium tellurite showed predominant activity against* Bacillus subtilis*,* Staphylococcus aureus*, and* Proteus vulgaris* [13]. As a continuation of earlier work, we determined to screen* sodium selenite* against selected human pathogenic bacteria. During the last few years several studies have been established to demonstrate* sodium selenite* as antioxidants and anticancer drugs [1, 2, 14]. The establishment of antibacterial screening of* sodium selenite* is very uncommon. In 2002 researcher has demonstrated that* sodium selenite* has bactericidal effect on* Helicobacter pylori* [12]. However, a report published in 2011 [9] showed that* sodium selenite* does not show any antibacterial effect on the species* Bacillus subtilis*,* Bacillus mycoides*,* Escherichia coli*, and* Pseudomonas *sp. In this work we screened the antibacterial effect of* sodium selenite* and the spectrum of activity was found to be predominant especially against* Bacillus subtilis* and* Klebsiella planticola *(Figure 1) and the least against* Streptococcus pyogenes* and* Pseudomonas aeruginosa *(Table 1) when compared to standard ciprofloxacin disc (5 *μ*g/disc). Moreover, in our previous study* Escherichia coli* and* Klebsiella planticola* were found to be more resistant against sodium tellurite. In this study* sodium selenite* was observed to be predominant zone of inhibition against both Gram positive and Gram negative bacteria. The result demonstrates that significant variations were observed on the efficacy of* sodium selenite* in Gram positive bacteria when compared to Gram negative bacteria (Table 2). As seen in Table 2,* sodium selenite* exhibited maximum activity against* B. subtilis* followed by* S. aureus*. Comparing the efficacy of* sodium selenite* between the cocci bacteria, predominant effect was observed against* S. aureus* and significantly lesser effect against* S. pyogenes*. This might be due to complex nature of cell wall of* S. pyogenes *when compared to* S. aureus* on comparing the efficacy of* S. pyogenes* with rod shaped* B. subtilis*. In this work it was observed that the spectrum of activity of* sodium selenite* expressed was slightly higher against* B. subtilis* when compared to* S. aureus*. Among Gram negative bacteria* sodium selenite* was found to be more effective against* K. planticola* and less effective against* P. aeruginosa *when compared with the standard ciprofloxacin. Comparing the efficacy of* sodium selenite* against Gram positive and Gram negative rod shaped bacteria, it is very interesting to note that* sodium selenite* exhibited broad spectrum of activity except* P. aeruginosa* (Table 3). In general, we observed that* sodium selenite* exhibits good spectrum of activity in mixed fashion and the results are more promising when compared with standard ciprofloxacin. However, it is an attempt to develop a new concept in inorganic element research to develop antibacterial substances which will lead to opening new concept in the antibacterial field.

## 5. Conclusion

In this preliminary study the results demonstrate that the* sodium selenite* is a promising candidate showing wider spectrum of activity against selected human pathogenic bacteria. However, further studies are under progress to find out the efficacy against various other pathogenic bacteria. Therefore, the study has to be focused further to get a clear conclusion that can be predicted to develop a new antibacterial agent since the problem of multiple drug resistance prevails for most of the available antibiotics.

## Figures and Tables

**Table 1:** Antibacterial effect of *sodium selenite* against selected human pathogenic bacteria.

Organisms	Zone of inhibition (mm) ± SEM	
	*Sodium selenite* (1% w/v)	Ciprofloxacin (5 mcg/disc)
*Streptococcus pyogenes*	15.5 ± 0.84^*∗∗∗*^	28 ± 2.6
*Staphylococcus aureus*	24 ± 0.73	26 ± 1.2
*Bacillus subtilis*	25.66 ± 0.49	24 ± 1.4
*Escherichia coli*	21.83 ± 0.47	21 ± 2.6
*Pseudomonas aeruginosa*	18.83 ± 1.07	20 ± 0.2
*Klebsiella planticola*	26.33 ± 1.11	23.5 ± 1.4

Each value is the mean of *n* = 6 batches with standard deviation; ^*∗∗∗*^ *P* < 0.001 is extremely significant when compared to standard drug by performing the Tukey Kramer test (*post hoc* analysis).

**Table 2:** Comparative study on the spectrum of activity among Gram positive and among Gram negative bacteria.

Comparison	Products	
	*Sodium selenite* (1% w/v)	Ciprofloxacin (5 *μ*g/disc)
Gram positive bacteria		
*S. pyogenes *versus* S. aureus*	^*∗∗∗*^ *P* < 0.001	*P* > 0.05, ns
*S. pyogenes *versus* B. subtilis*	^*∗∗*^ *P* < 0.01	*P* > 0.05, ns
*S. aureus *versus* B. subtilis*	^*∗∗*^ *P* < 0.01	*P* > 0.05, ns
Gram negative bacteria		
*E. coli *versus* P. aeruginosa*	*P* > 0.05, ns	*P* > 0.05, ns
*E. coli *versus* K. planticola*	^*∗*^ *P* < 0.05	^*∗*^ *P* < 0.05
*P. aeruginosa *versus* K. planticola*	^*∗∗∗*^ *P* < 0.001	^*∗*^ *P* < 0.05

^*∗∗∗*^Extremely significant (999 confidence interval); ^*∗∗*^extremely significant (99 confidence interval); ^*∗*^significant (95 confidence interval); ns (not significant).

**Table 3:** Gram positive versus Gram negative comparative study.

Comparison	Products	
	*Sodium selenite* (1% w/v)	Ciprofloxacin (5 *µ*g/disc)
*S. pyogenes *versus* E. coli*	^*∗*^ *P* < 0.05	^*∗∗∗*^ *P* < 0.001
*S. pyogenes *versus* P. aeruginosa*	*P* > 0.05, ns	^*∗∗∗*^ *P* < 0.001
*S. pyogenes *versus* K. planticola*	^*∗∗∗*^ *P* < 0.001	^*∗∗*^ *P* < 0.01
*S. aureus *versus* E. coli*	*P* > 0.05 ns	*P* > 0.05
*S. aureus *versus* P. aeruginosa*	^*∗∗*^ *P* < 0.01	*P* > 0.05 ns
*S. aureus *versus* K. planticola*	*P* > 0.05 ns	*P* > 0.05 ns
*B. subtilis *versus* E. coli*	*P* > 0.05 ns	*P* > 0.05 ns
*B. subtilis *versus* P. aeruginosa*	*P* > 0.05 ns	*P* > 0.05 ns
*B. subtilis *versus* K. planticola*	*P* > 0.05 ns	*P* > 0.05 ns

^*∗∗∗*^Extremely significant (999 confidence interval); ^*∗∗*^extremely significant (99 confidence interval); ^*∗*^significant (95 confidence interval); ns (not significant).
